# The predictors to medication adherence among adults with diabetes in the United Arab Emirates

**DOI:** 10.1186/s40200-016-0254-6

**Published:** 2016-08-09

**Authors:** Mohammed M. M. Al-Haj Mohd, Hai Phung, Jing Sun, Donald E. Morisky

**Affiliations:** 1 School of Public Health, Griffith University, Gold Coast, Australia; 2 Dubai Police Health Centre, Dubai, United Arab Emirates; 3 Department of Community Health Sciences, UCLA Fielding School of Public Health, Los Angeles, USA

**Keywords:** Diabetes, Adherence, Medication

## Abstract

**Background:**

Diabetes is a chronic medical condition and adherence to medication in adults with diabetes is important. Identifying predictors to medication adherence in adults with diabetes would help identify vulnerable patients who are likely to benefit by improving their adherence levels.

**Methods:**

We conducted a cross-sectional study at the Dubai Police Health Centre between February 2015 and November 2015. Questionnaires were used to collect socio-demographic, clinical and disease related variables and the primary measure of outcome was adherence levels as measured by the Morisky Medication Adherence Scale (MMAS-8©). Multivariate logistic regression was carried out to identify predictors to adherence.

**Results:**

Four hundred and forty six patients were interviewed. Mean age 61 year +/− 11. 48.4 % were male. The mean time since diagnosis of diabetes was 3.2 years (Range 1–15 years). Two hundred and eighty eight (64.6 %) patients were considered non-adherent (MMAS-8© adherence score < 6) while 118 (26.5 %) had moderate adherence (MMAS-8© adherence score 6 = <8) and 40 (9.0 %) high adherence (MMAS-8© adherence scores <8) to their medication respectively. The strongest predictor for adherence as predicted by the multi-logistic regression model was the patient’s level of education. A technical diploma certificate as compared to a primary school level of education was the strongest predictor of adherence (OR = 66.1 CI: 6.93 to 630.43); *p* < 0.001). The patient’s age was also a predictor of adherence with older patients reporting higher levels of adherence (OR = 1.113 (CI: 1.045 to 1.185; *p* = 0.001 for every year increase in age). The duration of diabetes was also a predictor of adherence (OR = 1.830 (CI: 1.270 to 2.636; *p* = 0.001 for every year increase in the duration of diabetes). Other predictors to medication adherence include Insulin use, ethnicity and certain cultural behaviours.

**Conclusion:**

A number of important predictors to medication adherence in diabetics were identified in this study. Such predictors could help develop policies for improving adherence in diabetics.

## Background

Diabetes mellitus is a chronic metabolic disease affecting approximately 341 million to 371 million people worldwide [1, 2]. Furthermore, it is estimated that one third of those affected (approximately 122.5 million) are not aware that they have the condition [3].

The oil boom has led to a massive increase in the GDP and disposable income of the people of the United Arab Emirates (UAE). The UAE was ranked as the 19th highest income countries of the world in 2012 (International Monetary Fund) and is categorized as a high income country according to the (World bank, 2012). This has led to a more affluent lifestyle, and from health point an increase in total calorie intake per person together with a decrease in calorie expenditure. This has led to a nationwide obesity pandemic with the rates of obesity climbing to record highs and standing at about 68 % of the population according to one estimate from 2007.

The United Arab Emirates (UAE) is now being listed as the country with the 11th highest prevalence of diabetes globally (primarily type-2) [4]. Furthermore, metabolic control of diabetes is reportedly poor, leading to an increased risk of associated complications [5]. Almost 70 % of Emirati nationals are reported to be overweight or obese [6], and one third of Emirati children are also now obese [7]; these figures are two to three times those of international standards [8], thus, it is not surprising that the prevalence of type-2 diabetes has escalated.

Medication non-adherence is of increasing concern for healthcare providers despite the known benefits of modern treatment regimens, with prevalence reported in one study to be in excess of 50 % of diabetic patients. The consequences of non-adherence include not only health-related consequences (i.e. failure of treatment, re-hospitalisation, death), but also financial consequences as the cost of emergency medical interventions as a consequence of non-adherence outweigh the combined cost of an adhered-to medication regimen [9]. The WHO has identified non-adherence as a multifactorial problem caused by the interplay of factors from any of the following 5 areas: 1) the patient, 2) the condition, 3) the type of therapy prescribed, 4) socioeconomic factors, and 5) health system related factors [10]. Several studies have been carried out looking at medication adherence in diabetic patients around the world; however no studies have been performed in the U.A.E [11–13].

## Methods

### Study design

This was a cross-sectional study looking at predictors to medication adherence among diabetics as measured by the Morisky Medication adherence scale (MMAS-8©). The predictors were identified from variables collected via questionnaires and then analysed using statistical software.

### Study setting

The study took place at the Dubai Police Health Services Clinic between February 2015 and November 2015. This centre provides primary care and speciality care for all Dubai Police employees and members of their families and had over 200,000 clinic visits in 2014 alone. The patients were identified from the diabetes clinics and were approached at the time of their routine scheduled diabetes checks. The patients were given a patient information sheet and the study was explained to them. The patients were then asked to sign a consent sheet and subsequently asked to fill in a questionnaire and attend a blood test. The research topic was granted an ethical clearance through the Human Research Ethics Committee at Griffith University GU Ref No: PBH/11/14/HREC and confirmed with the Dubai Police Research Ethics standards.

### Recruitment procedure (inclusion and exclusion criteria)

All patients had to be type 2 diabetics on at least one anti-diabetic medication following a diabetes diagnosis of one year or more. Male and female patients between the age of 18 and 80 years were eligible for the study. The following exclusions applied: cognitive impairment (if previously included in medical history),, pregnant or breastfeeding women, non-residents and patients who did not comprehend either the English or Arabic language.

The investigators identified and screened potential participants in the following manner: all patients present in the waiting area of the diabetic clinic were screened by reviewing their medical charts to ensure that all inclusion criteria were met. They were then asked if they are willing to talk to the investigator. If the person agreed an informed consent was then read and explained by the investigators. Once verbal consent was obtained, the inclusion and exclusion criteria were checked again. The Tablet based questionnaires required for the study were explained to the patient and a qualified phlebotomist obtained the blood sample. All participants completed the questionnaires in a private clinic room.

### Data collection techniques

Data was collected primarily via questionnaires. These questionnaires were written using the Android Database software package (MEMENTO). The questionnaires were then handed out in an electronic form using an Android tablet. The tablets were purchased for the purpose of this research and were solely used for this purpose. These tablets were secured with an encrypted password and were locked in the research office when not in use. The tablets and questionnaires were supervised by one of the research investigators at the time of interview to ensure the correct handling of the questionnaire and to troubleshoot any problems if they would arise. The questionnaire forms were based on a number of field styles including free text, date, single best answer and multi-check selections. The database software collated the results into an excel sheet which was later exported to a computer for further data analysis.

### Data variables

The data collated via the questionnaires included the following variables: age, gender, ethnicity, marital status, highest level of education attained, working conditions, transport availability, smoking status, diabetes duration, cultural factors (dress wear, behaviors in Ramadan, perception towards obesity), number of antidiabetic medication, insulin therapy, the Depression, Anxiety and Stress scale (DASS-21) and International Physical Activity Questionnaire (IPAQ) score. The Morisky Medication Adherence Scale −8 (MMAS-8) score was used to measure the outcome of adherence to medication. MMAS-8. Glycated hemoglobin (HBA1c (%)) was used to check the validity of the MMAS-8 score in this cohort as a measure of adherence to medication among the study participants.

### Morisky medication adherence scale

The Morisky Medication Adherence Scale (MMAS) was designed to determine adherence behaviours [14]. For this study the 8-item model of this scale was used; patients were asked 8 questions designed to determine which factors affect how well they adhered to their medication regimen Table 1.

**Table 1:** The 8 questions asked to determine medication adherence based on the Morisky Medication Adherence Scale

Item	Answer	Number	%
Do you sometimes forget to take your diabetes pills?	Yes	176	39.5 %
	No	270	60.5 %
People sometimes miss taking their medications for reasons other than forgetting. Thinking over the past two weeks, were there any days when you did not take your Diabetes medicine?	Yes	169	37.9 %
	No	277	62.1 %
Have you ever cut back or stopped taking your medication without telling your doctor, because you felt worse when you took it?	Yes	149	33.4 %
	No	297	66.6 %
When you travel or leave home, do you sometimes forget to bring along your Diabetes medication?	Yes	220	49.3 %
	No	226	50.7 %
Did you take your Diabetes medicine yesterday?	No	388	87.0 %
	Yes	58	13.0 %
When you feel like your diabetes is under control, do you sometimes stop taking your medicine?	Yes	261	58.5 %
	No	185	41.5 %
Taking medication everyday is a real inconvenience for some people. Do you ever feel hassled about sticking to your diabetes treatment plan?	Yes	204	45.7 %
	No	242	54.3 %
How often do you have difficulty remembering to take all of your medicine?	Never/rarely	50	11.2 %
	Once in a while	69	15.5 %
	Sometimes	96	21.5 %
	Usually	101	22.6 %
	All the time	130	29.1 %

Patients are required to answer the questions with either a ‘yes’ or a ‘no’, with the final question taking the form of a typical five-point Likert item. Positive answers (i.e. yes) are scored a 1 and negative answers (i.e. no) are scored a 0. From these responses a final score was calculated with three possible outcomes; a score of >2 corresponded to low medication adherence, a score of 1 or 2 corresponded to medium medical adherence, and a score of 0 corresponded to high medical adherence. The MMAS is a popular, easy and economical method of data collection, facilitating the collection of a large amount of data in a short period of time [15].

### Depression, anxiety and stress scale

The Depression, Anxiety and Stress scale (DASS) was developed by researchers in New South Wales, Australia. Its purpose is to measure the patient’s response to a series of questions regarding the frequency and severity of recent negative, emotional experiences. In total, there are 42 items (although the DASS is also available in a condensed 21 item form the DASS 21) to which patients are required to answer via the means of a four-point Likert scale. Each category (depression, anxiety and stress) is examined by 14 items each, further broken down into subcategories to examine specific aspects of each condition. The score (sum or mean) of individual scales (depression, anxiety OR stress), or the total scale (depression, anxiety AND stress) can be used to provide an indication of the severity of the patient’s condition. As depression, anxiety and stress are intrinsically dimensional, assigning a ‘cut-off’ or ‘clinical norm’ against which to compare patients, is arbitrary. However, the outcome of the DASS has been reported as normal, mild, moderate, severe, or extremely severe [16], The DASS has been fully validated against a number of existing measures of depression, anxiety and stress [17–19]. The DASS-21 score was utilized in this study.

### International physical activity questionnaire

The IPAQ score has been extensively validated and culturally adopted and translated into different languages including Arabic. The questionnaires used for employed the “7-day recall IPAQ self-administered version”. The Arabic translation for these items has been published online and has been used in different research in Arabic speaking countries [20, 21]. The Arabic translation for these items has been published online and has been used in different research in Arabic speaking countries. The IPAQ score measured would be a useful way of measuring activity in the study population in a standardized method which would increase the reliability of the data collected and, in the future, make it easier to compare findings from this study with others. The IPAQ score protocol used to analyse the questionnaire has been published online by the official IPAQ group on the following webpage (www.IPAQ.ki.se) and is summarized as follows:

Category 1: Low This is the lowest level of physical activity. Those individuals who do not meet criteria for categories 2 or 3 are considered low/inactive. Category 2: Moderate Any one of the following 3 criteria:

• 3 or more days of vigorous activity of at least 20 min per day OR • 5 or more days of moderate-intensity activity or walking of at least 30 min per day OR • 5 or more days of any combination of walking, moderate-intensity or vigorous intensity activities achieving a minimum of at least 600 MET-min/week. Category 3: High Any one of the following 2 criteria: • Vigorous-intensity activity on at least 3 days and accumulating at least 1500 MET-minutes/week OR • 7 or more days of any combination of walking, moderate-intensity or vigorous intensity activities achieving a minimum of at least 3000 MET-minutes/week.

## Results

**Fig. 1:**
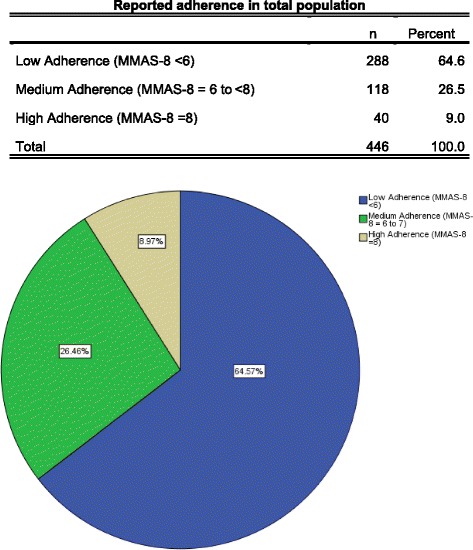
Reported adherence rates in total study population

The Baseline characteristics are shown in Table 2. Two hundred and eighty eight (64.6 %) patients were considered non-adherent (MMAS-8© adherence score < 6) while 118 (26.5 %) and 40 (9.0 %) had medium adherence (MMAS-8© adherence score 6 to <8) and high adherence (MMAS-8© adherence scores =8) to their medication respectively (Fig. 1).

**Table 2:** Self-reported medication adherence behaviour of study participants as determined by the Morisky 8-Item Medication Adherence Scale (MMAS-8©)

Age (mean +/− std)		61 +/− 11
Gender	Female	51.6 % (230)
	Male	48.4 %(216)
Ethnicity	Arab Emarati	56.1 % (250)
	Arab Non-Emarati	38.1 % (170)
	Asian	5.8 % (26)
HbA1c baseline (mean +/− std)		8.50 +/− 0.07
SBP at baseline (mean +/− std)		133 +/− 26
DBP at baseline (mean +/− std)		72 +/− 21
HDL at baseline (mean +/− std)		54 +/− 11
LDL at baseline (mean +/− std)		129 +/− 37
TGL at baseline (mean +/− std)		212 +/− 42
Anti-Diabetic therapy	Monotherapy	29.4 % (131)
	Combination	70.6 % (315)
Insulin use	Yes	50.2 % (224)
	No	49.8 % (222)
Prescence of chronic conditions	Yes	54.0 % (224)
	No	46.0 % (205)

More than a third (39.5 %) of the participants reported that sometimes they forgot to take their anti-diabetic medications; 37.9 % reported that they did not take their medications on at least one occasion in the two weeks before the interview; 33.4 % reported that they discontinued taking their medications without telling their doctor when they felt worse upon taking their medications; 49.3 % reported that they sometimes forgot to take their medications with them when they travelled or left home; 13.0 % reported taking all their medications on the day before the interview; 58.5 % reported that they stopped taking their medicines when they felt like their diabetic symptoms were under control; 45.7 % reported feeling hassled by their treatment plans; and finally 88.8 % reported that they had difficulty remembering to take all their medicines at least once in awhile (Table 1).

### Reliability, internal consistency and validity of MMAS-8

The Cronbach’s alpha test was calculated for the 8-item MMAS-8 and this was reliable at 0.736 Omission of any of the 8-items of the MMAS-8 questionnaire resulted in a lower Cronbach’s alpha. The validity of MMAS-8 adherence score was assessed by testing the ability of the score to distinguish between groups of individuals that differ from each other according to the HbA1c. There was a significant difference in the Mean HbA1c levels among the three adherence groups. Mean HbA1c was 9.24, 7.33 and 6.60 % in the low, medium and high adherence groups respectively (*p* < 0.05).

### Patient, socioeconomic and health care system factors

#### Age, gender and ethnicity

There was a statistically significant difference in the mean age of patients who reported low adherence levels (MMAS-8 < 6) compared to those who reported Medium (MMAS-8 = 6 to 7) or High adherence levels (MMAS-8 = 8) (59 years versus 64 years, 69 years respectively *p* < 0.05).

Females tended to report high adherence levels compared to males (13.5 % vs 4.2 %, *p* = 0.001). Adherence levels also differed significantly between different ethnic groups with the lowest adherence levels reported by Emirati patients (81.6 %) followed by Arab Non-Emirati (47.1 %) and Asians (15.4 %) (Pearson Chi-Square *p* < 0.001) (Table 3).

**Table 3:** Adherence levels differed significantly with the patients gender and ethnic group

		Adherence level					
		Low adherence (MMAS-8 < 6)		Medium adherence (MMAS-8 = 6 to <8)		High adherence (MMAS-8 = 8)	
		Row %	N	Row %	n	Row %	n
Gender	Female	57.8 %	133_a_	28.7 %	66_a_	13.5 %	31_b_
	Male	71.8 %	155_a_	24.1 %	52_a_	4.2 %	9_b_
Ethnicity	Arab Emarati	81.6 %	204_a_	15.6 %	39_b_	2.8 %	7_b_
	Arab Non-Emarati	47.1 %	80_a_	38.2 %	65_b_	14.7 %	25_b_
	Asian	15.4 %	4_a_	53.8 %	14_b_	30.8 %	8_b_

*Note*: Values in the same row and sub-table not sharing the same subscript are significantly different at *p* < .05 in the two-sided test of equality for column proportions. Cells with no subscript are not included in the test

### Marital status, living arrangements, level of education, working status and transport availability

Married patients represented the majority of the cohort group with 66 % (*n* = 254), 26.5 % (*n* = 102), and 7.5 % (*n* = 29) of the patients married in the low, medium and high adherence groups respectively. Seventy-One per cent (*n* = 5) of widowed patients reported high levels of adherence (MMAS-8 = 8) compared to 28.6 % (*n* = 2) who reported medium or low levels of adherence. This was a statistically significant difference (*p* < 0.001), however of note is the small number of overall widowed patients. Patients who reported high adherence levels (MMAS-8 = 8) were more likely to have a higher education level compared to those who reported medium or low adherence rates; 62.5 % had a university degree vs 21.2 and 15.6 % (*p* < 0.005). There were no significant differences in the living arrangements, transportation availability or working status between the different adherence groups.

### IPAQ

Two-hundred and sixty eight patients reported low physical activity as indicated by their IPAQ scores. Low physical activity was reported more in the group of low adherence as compared to medium and high adherence patients (75.7 % vs 22.4 % vs 1.9 % *p* < 0.001). Conversely, patients with higher physical activity scores reported higher adherence levels.

### DASS-21

The DASS-21 score was calculated for each of the following categories; depression, anxiety and stress. Low adherents to medication were less likely to report a normal depression score compared to those who were moderately and highly adherent to their medication (39.6 % vs 49.2 % vs 70 % respectively, *p* = 0.004). Low adherents to medication were also more likely to report severe levels of anxiety scores compared to those who were moderately and highly adherent to their medication (29.2 % vs 16.9 % vs 10.0 % respectively, *p* < 0.001). Low adherents to medication were also more likely to report severe stress scores compared to those who were moderately and highly adherent to their medication (20.1 % vs 8.5 % vs 5.0 % respectively, *p* = 0.004).

### Cultural factors

Statistical analysis of the data collected has revealed interesting and novel findings when it comes to the influence of cultural factors towards the attitude of diabetic patients to their adherence to medication (Table 4). Low adherents to medication were more likely to wear loose traditional clothes (Dishdasha, Kandoura, Abaya and Jelbab) compared to those who were moderately and highly adherent to their medication (75.7 % vs 19.5 % vs 57.5 % respectively, *p* < 0.001). Low adherents to medication were also less likely to alter their medication under the guidance of their doctor during fasting in the Holy month of Ramadan compared to those who were moderately and highly adherent to their medication (92 % vs 53.4 % vs 32.5 % respectively, *p* < 0.001). Low adherents to medication were also less likely to notice changes in their weight compared to those who were moderately and highly adherent to their medication (85.1 % vs 62.7 % vs 30 % respectively, *p* < 0.001). Low adherents to medication were more likely to gain weight during the fasting month of Ramadan compared to those who were moderately and highly adherent to their medication (83.7 % vs 48.3 % vs 32.5 % respectively, *p* < 0.001). Low adherents to medication were more likely to find hot weather during summer a challenge to exercise compared to those who were moderately and highly adherent to their medication (88.2 % vs 39.8 % vs 47.5 % respectively, *p* < 0.001).

**Table 4:** Cultural variables in relation to adherence levels

		Adherence level					
		Low adherence (MMAS-8 < 6)		Medium adherence (MMAS-8 = 6 to 7)		High adherence (MMAS-8 = 8)	
		n	Column %	n	Column %	n	Column %
What do you mostly wear?	Shirt, trousers, shorts, skirt.	70_a_	24.3 %	95_b_	80.5 %	17_c_	42.5 %
	Dishdasha/Abaya/Kandoura/Jelbab	218_a_	75.7 %	23_b_	19.5 %	23_c_	57.5 %
Do you alter your medication in Ramadan under the guidance of your doctor?	Yes	23_a_	8.0 %	55_b_	46.6 %	27_b_	67.5 %
	No	265_a_	92.0 %	63_b_	53.4 %	13_b_	32.5 %
Do you notice any changes in your weight?	Yes	43_a_	14.9 %	44_b_	37.3 %	28_c_	70.0 %
	No	245_a_	85.1 %	74_b_	62.7 %	12_c_	30.0 %
Do you gain or lose weight in Ramadan?	Lose weight	40_a_	13.9 %	44_b_	37.3 %	20_b_	50.0 %
	Stay the same	7_a_	2.4 %	17_b_	14.4 %	7_b_	17.5 %
	Gain weight	241_a_	83.7 %	57_b_	48.3 %	13_b_	32.5 %
Do you see plumpness as a sign of beauty?	Yes	150_a_	52.1 %	52_a_	44.1 %	15_a_	37.5 %
	No	138_a_	47.9 %	66_a_	55.9 %	25_a_	62.5 %
Do you find hot weather (Summer) a challenge to exercise?	Yes	254_a_	88.2 %	47_b_	39.8 %	19_b_	47.5 %
	No	34_a_	11.8 %	71_b_	60.2 %	21_b_	52.5 %

*Note*: Values in the same row and sub-table not sharing the same subscript are significantly different at *p* < .05 in the two-sided test of equality for column proportions. Cells with no subscript are not included in the test. Tests assume equal variances^1^

1. Tests are adjusted for all pairwise comparisons within a row of each innermost sub-table using the Bonferroni correction

### Condition and therapy variables

Low adherents to medication were less likely to be on combination anti-diabetic therapy compared to those who were moderately and highly adherent to their medication (68.1 % vs 89.8 %, *p* < 0.001). Low adherents to medication were also more likely to use Insulin compared to those who were moderately and highly adherent to their medication (58.7 % vs 37.3 % vs 27.5 % respectively, *p* < 0.001). Low adherents to medication were also less likely to have other chronic conditions compared to those who were moderately and highly adherent to their medication (46.9 % vs 64.4 % vs 75.0 % respectively, *p* < 0.001) Table 5.

**Table 5:** Condition and therapy variables against different reported adherence levels

		Adherence level					
		Low adherence (MMAS-8 < 6)		Medium adherence (MMAS-8 = 6 to 7)		High adherence (MMAS-8 = 8)	
		n	Column %	n	Column %	n	Column %
Anti-diabetic therapy	Monotherapy	92_a_	31.9 %	12_b_	10.2 %	27_c_	67.5 %
	Combination	196_a_	68.1 %	106_b_	89.8 %	13_c_	32.5 %
Insulin use	Yes	169_a_	58.7 %	44_b_	37.3 %	11_b_	27.5 %
	No	119_a_	41.3 %	74_b_	62.7 %	29_b_	72.5 %
Presence of chronic conditions	Yes	135_a_	46.9 %	76_b_	64.4 %	30_b_	75.0 %
	No	153_a_	53.1 %	42_b_	35.6 %	10_b_	25.0 %

Note: Values in the same row and sub-table not sharing the same subscript are significantly different at *p* < .05 in the two-sided test of equality for column proportions

Low adherents to medication had shorter mean duration of diabetes compared to those who were moderately and highly adherent to their medication (Mean 2 years vs 4 years vs 7 years respectively, *p* < 0.05).

### Multi-logistic regression analysis

The following points were undertaken during multilogistic regression analysis. Adherence was re-classified into adherents (MMAS-8 > 6) and non-adherents groups (MMAS-8 < 6).

Multilogistic regression performed using IBM SPSS Version 20 with all the variables described above. Cox & Snell R square = 0.660 and Nagelkerke R Square = 0.908; indicating good fitness of model.

**Fig. 2:**
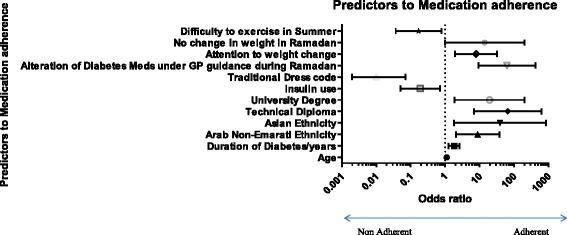
Multi-variate logistic regression of predictors to medication adherence

The strongest predictor for adherence as predicted by the multi-logistic regression model was the patient’s level of education. A technical diploma certificate as compared to a primary school level of education was the strongest predictor of adherence (OR = 66.1 CI: 6.93 to 630.43); *p* < 0.001). The patient’s age was also a predictor of adherence with older patients reporting higher levels of adherence (OR = 1.113 (CI: 1.045 to 1.185; *p* = 0.001 for every year increase in age). The duration of diabetes was also a predictor of adherence (OR = 1.830 (CI: 1.270 to 2.636; *p* = 0.001 for every year increase in the duration of diabetes). The patient’s ethnicity was also a predictor of adherence with Arab Non-Emirati and Asian ethnicities predicting a higher level of adherence compared with Emirati ethnicity (OR = 8.830 (CI: 2.052 to 37.995) *p* = 0.003; OR = 39.4 (CI: 1.819 to 853.46) *p* = 0.19 respectively). University level of education was a predictor for adherence compared to primary/secondary school level of education (OR = 19.6 (CI: 1.872 to 205.130); *p* = 0.013). The behaviour of altering one’s medication under the guidance of their doctor during the fasting month of Ramadan was a strong predictor of adherence as well (OR = 62.68 (CI: 9.324 to 421.286; *p* < 0.001). The behaviour of patients around paying attention to one’s weight was a predictor of adherence as well (OR = 7.965 (CI: 1.971 to 32.18; *p* = 0.004). Conversely, the challenge of exercising due to hot weather in summer was a predictor of non-adherence (OR = 0.170 (CI: 0.037 to 0.777) *p* = 0.022). Similarly insulin use and traditional Arabic dress code were associated with non-adherence (OR = 0.188 (CI: 0.05 to 0.709); *p* = 0.014; OR = 0.010 (CI: 0.002 to 0.071) *p* = 0.188 respectively) (Table 6, Fig. 2).

**Table 6:** Significant predictors to adherence after multi-logistic regression modelling

	p-value	Odds ratio	95 % C.I. for OR	
			Lower	Upper
Age	.001	1.113	1.045	1.185
Duration of diabetes/years	.001	1.830	1.270	2.636
Arab Non-Emarati ethnicity	.003	8.830	2.052	37.995
Asian ethnicity	.019	39.400	1.819	853.457
Technical diploma	.000	66.076	6.925	630.433
University degree	.013	19.596	1.872	205.130
Insulin use	.014	.188	.050	.709
Traditional dress code	.000	.010	.002	.071
Alteration of diabetes Meds under GP guidance during Ramadan	.000	62.675	9.324	421.286
Attention to weight change	.004	7.965	1.971	32.180
No change in weight in Ramadan	.050	14.112	.995	200.233
Difficulty to exercise in summer	.022	.170	.037	.777

## Discussion

Limited data exist on the adherence of diabetics in the United Arab Emirates to their medication prescribed by their doctors as well as on the factors influencing their adherence. Studies in other countries have demonstrated poor adherence rates of medication among diabetics and patients suffering with other chronic conditions. Al Mazroui demonstrated a significant reduction in the levels of HbA1c among diabetics receiving an intensive educational program over a 12 month period of time (baseline vs. 12 months; 95 % confidence interval) of HbA1c8.5 % (8.3, 8.7) vs. 6.9 % (6.7, 7.1) [22]. Reed demonstrated the important role of chronic diabetes clinics in the UAE at improving diabetes outcomes as measured by HbA1c levels and blood pressure [23].

Despite this lack of data from the United Arab Emirates there have been numerous studies from around the world looking at the impact of medication adherence on outcome in patients of chronic medical diseases including diabetes. An American observational study concluded that high adherence levels to medication among diabetics were associated with an overall reduction in healthcare costs [24]. Another retrospective study of over 11,000 patients showed that poorly non -adherent diabetics had higher all-cause hospitalization and all-cause mortality compared to adherent diabetics [9]. Adherence to medication in diabetes is therefore of utmost importance, and identifying factors that lead to poorer medication compliance should be identified to guide healthcare policy.

The adherence rates among diabetics in this group of type 2 diabetics followed through a primary care setting were low (64.6 % of patients were considered non-adherent with a MMAS-8© adherence score < 6). The predictors of nonadherence to medications were studied using a multi-logistic regression model and different socio-economic, demographic and disease related factors were identified. The strongest predictor for adherence as predicted by the multi-logistic regression model was the patient’s level of education. A technical diploma certificate as compared to a primary school level of education was the strongest predictor of adherence (OR = 66.1 CI: 6.93 to 630.43); *p* < 0.001). This finding of lower adherence levels in those with a lower education achievement should shift focus on this group with greater levels of care directed to them to ensure the issue of adherence is addressed.

The patient’s ethnicity was also found to be a predictor of lower adherence levels, with the local Emirati population having a lower level of adherence. Non-Emirati patients tend to be a highly skilled workforce reflecting a higher level of education and skills. This could explain the higher level of understanding of their underlying medical condition and the importance of medication adherence.

The patient’s age was also a predictor of adherence with older patients reporting higher levels of adherence (OR = 1.113 (CI: 1.045 to 1.185; *p* = 0.001 for every year increase in age). The duration of diabetes was also a predictor of adherence (OR = 1.830 (CI: 1.270 to 2.636; *p* = 0.001 for every year increase in the duration of diabetes). Subsequently, younger patients with a shorter duration of diabetes are less likely to have come to terms with the diagnosis of their diabetes and had a shorter time to grasp the concept of medication adherence than their older counterparts. This group of patients tend to be more vulnerable and therefore require more policies to be in place to ensure they are well supported and encouraged during this early phase of diagnosis.

The behaviour of altering one’s medication under the guidance of their doctor during the fasting month of Ramadan was a strong predictor of adherence as well (OR = 62.68 (CI: 9.324 to 421.286; *p* < 0.001). The behaviour of patients around paying attention to one’s weight was a predictor of adherence as well (OR = 7.965 (CI: 1.971 to 32.18; *p* = 0.004). Conversely, the challenge of exercising due to hot weather in summer was a predictor of non-adherence (OR = 0.170 (CI: 0.037 to 0.777) *p* = 0.022). Similarly insulin use and traditional Arabic dress code were associated with non-adherence (OR = 0.188 (CI: 0.05 to 0.709); *p* = 0.014; OR = 0.010 (CI: 0.002 to 0.071) *p* = 0.188 respectively).

Non-adherence may arise as a consequence of the patient knowingly disregarding their treatment regimen (active non-adherence), or as a consequence of carelessness or forgetfulness, whereby patients occasionally omit their medication from their daily routine or take that medication later than required (passive non-adherence).

The limitations of this study include the small size, which despite meeting the pre-determined study size sample predicted before starting the study would continue to be a source of population bias error. The MMAS is a popular, easy and economical method of data collection, facilitating the collection of a large amount of data in a short period of time. Furthermore, the questions are purposely phrased to avoid the ‘yes-saying’ bias as it is known that patients feel they should provide healthcare providers with a positive response. However, there are limitations to this method of data collection; the MMAS does not account for personal or lifestyle factors (e.g. age, physical ability, means of transport, known methods of communication, etc.), and the outcome of these questions can be biased by patients supplying false information. There was no direct measurement of adherence to medication however the MMAS-8 score has been well validated in measuring medication adherence in diabetes and other chronic conditions.

## Conclusion

Adherence to medication among diabetics continues to be low. A number of important predictors to medication adherence in diabetics were identified in this study. Such predictors could help develop policies for improving adherence in diabetics.

## Abbreviations

DASS, depression, anxiety and stress scale; HbA1c, Glycated Haemoglobin alpha concentration; IPAQ, international physical activity questionnaire; MMAS-8, Morisky medication adherence scale- 8; U.A.E., United Arab Emirates; WHO, World Health Organisation
